# Clinical and genomic features of *Corynebacterium macginleyi*-associated infectious keratitis

**DOI:** 10.1038/s41598-021-85336-w

**Published:** 2021-03-16

**Authors:** Susanna Sagerfors, Anja Poehlein, Mastaneh Afshar, Birgitta Ejdervik Lindblad, Holger Brüggemann, Bo Söderquist

**Affiliations:** 1 Department of Ophthalmology, Faculty of Medicine and Health, Örebro University, 70182 Örebro, Sweden; 2 Department of Genomic and Applied Microbiology, Institute of Microbiology and Genetics, University of Göttingen, Göttingen, Germany; 3 Department of Biomedicine, Aarhus University, Aarhus, Denmark; 4 Department of Laboratory Medicine, Clinical Microbiology, Faculty of Medicine and Health, Örebro University, 70182 Örebro, Sweden

**Keywords:** Microbiology, Diseases, Molecular medicine

## Abstract

Infectious keratitis is a potentially sight threatening ophthalmological emergency. Contact lens wear is a common risk factor. Diagnostic advances such as MALDI-TOF MS provides new insights into the spectrum of corneal pathogens and on microbes previously considered as commensals. *Corynebacterium macginleyi* was described in 1995, and in 2018, the genomic features of three isolates were reported after whole-genome sequencing. Here we describe the clinical characteristics of patients with infectious keratitis (n = 29) presumably caused by *Corynebacterium macginleyi*, and analyze the genomic features of *C. macginleyi* (n = 22) isolated from the corneal ulcers of these patients. The disease course was uneventful apart from minor interventions such as corneal cross-linking and amniotic membrane transplant. Genome sequencing and comparison revealed a highly conserved core genome of *C. macginleyi*. Based on the analyses of single nucleotide polymorphisms, the population could be divided into two main clades that also differed in a few clade-specific genomic islands. Patients infected with an isolate belonging to the minor clade (n = 7) presented a more severe disease. Comparisons with other corynebacterial species clearly separated *C. macginleyi*. *C. macginleyi* may be considered a corneal pathogen; genomic analysis provided insights into its population structure and disease-causing potential.

## Introduction

*Corynebacterium macginleyi*, a slow-growing, lipid-requiring, Gram-positive, facultative anaerobic rod named after Kenneth McGinley, was first described by Riegel et al.^1^. All three strains of *C. macginleyi* covered in that report were isolated from human eyes, and from a phylogenetic perspective showed most resemblance to *Corynebacterium accolens*. With the exception of a few case reports of infections caused by *C. macginleyi*, such as endocarditis^2^, septicemia^3^, and infections related to foreign bodies including intravenous catheter infections^4^, bladder catheter infections^5^, ventilator-associated pneumonia^6^, and surgical site infections^7^ most reports on *C. macginleyi* are ocular infections. In the eye, *C. macginleyi* is known to cause conjunctivitis^8,9^, keratitis^10–12^, blebitis^13^, and endophthalmitis^14^. In 2000, Joussen et al. proposed that *C. macginleyi* is a conjunctival-specific pathogen^9^ that might benefit from the presence of the meibomian glands^15^. The meibomian glands, located in the tarsal plates, secrete the sterol and wax esters, polar lipids, triglycerides, free fatty acids, and di- and tri-esters that constitute the outer lipid layer of the tear film^16,17^. The lipid-rich environment of the ocular surface may offer an explanation for why lipophilic *C. macginleyi* colonizes the eye.

The first *C. macginleyi* isolates were whole-genome sequenced by Bernier and Bernard^18^. These three isolates showed an average G + C content of 57.1%, which is similar to the 58% reported by Riegel^1^, and an average of 2321 coding DNA sequences (CDS)^18^. In two recent reports on the microbiological profile of infectious keratitis in Portugal, *C. macginleyi* was the most common or second most common isolated agent: 18.41% (88/478) and 20% (13/65), respectively^10,19^.

The aim of the present study was to describe clinical characteristics of patients with infectious keratitis caused by *C. macginleyi,* and to examine the genomic traits of clinical isolates of *C. macginleyi* in order to evaluate the potential of this bacterial species to cause infectious keratitis.

## Results

We identified 29 patients from January 2004 to June 2018 with suspected infectious keratitis whose corneal cultures displayed growth of *C. macginleyi*. Median age was 55 years, and the most common risk factors for infectious keratitis were contact lens wear (19/29 cases; 66%) and ocular surface disease (3/29 cases; 10%) (Table 1). Polymicrobial growth was seen in 13 (45%) of the 29 patients (Table S1). All but one patient (28/29; 97%) fulfilled the clinical criteria for infectious keratitis^20^. The patient who did not fulfill the clinical criteria was a contact lens wearer (monthly replaceable contact lenses with extended wear, i.e. continuous wear both day and night) with two weeks of pain and redness in one eye. The onset of ocular symptoms was preceded by a common cold. The patient had continued to wear contact lenses until two days before the first consultation. At the first visit, the best corrected visual acuity was 0.4 Snellen decimals. Examination in a slit lamp revealed a mixed conjunctival injection and multiple infiltrates, both centrally and in the periphery, with stromal thickening and epithelial edema. No epithelial defect could be detected with fluorescein staining. After corneal culture, treatment was initiated with topical levofloxacin (5 mg/mL) hourly with an addition of ciprofloxacin ointment (3 mg/g) for the night when the levofloxacin treatment was tapered during night time. At the first follow up 2 days after the first visit, the patient’s discomfort had decreased, the conjunctival injection was diminished, the corneal infiltrates were thinner and smaller and the epithelial edema was reduced and so was the stromal thickening. Best corrected visual acuity was 0.7 Snellen decimals. The levofloxacin treatment was gradually tapered, no topical steroid or general pharmacological therapy were prescribed and no surgical intervention was performed. At follow up visit number 4 (the last visit, 16 days after the first visit), the patient was asymptomatic. Best corrected visual acuity was 1.0 Snellen decimals, and examination in a slit lamp revealed no conjunctival injection; instead subepithelial haze/scarring with some thinning of the stroma could be observed. The topical antibiotic treatment was discontinued after 17 days of treatment.

**Table 1:** Clinical characteristics of patients with *Corynebacterium macginleyi*-associated infectious keratitis, January 2004 to June 2018.

	Total (n = 29)	Monomicrobial growth (n = 16)	Polymicrobial growth (n = 13)	*p* value	Clade I (n = 15)	Clade II (n = 7)	*p* value
Median age at episode onset (25th percentile; 75th percentile)	55.0 (36.5;65.0)	53.5 (34.3;59.8)	55.0 (38.0;75.0)	0.469^d^	50.0 (41.0;57.0)	71.0 (63.0;79:0)	0.002^d^
Sex (female)	14	9	5	0.340^e^	8	3	1.0^f^
Laterality (right)	14	8	6	0.837^e^	7	5	0.381^f^
Contact lens wear	19	12	7	0.270^f^	11	2	0.074^f^
No known risk factors	3	1	2	0.573^f^	1	2	0.227^f^
Ocular surface disease including eyelid disorders	3	2	1	1.0^f^	1	1	1.0^f^
Corneal transplant	2	1	1	1.0^f^	1	1	1.0^f^
Combination of two of the above risk factors	2	0	2	0.192^f^	1	1	1.0^f^
Median duration of symptoms at first visit in days (25th percentile; 75th percentile)^a^	3.0 (2.0;6.25)	3.0 (1.8;3.3)	4.0 (2.3;13.0)	0.123^d^	3.0 (1.0:3.5)^a^	6.0 (2.0;14.0)	0.084^d^
Inpatient treatment	5	1	4	0.144^f^	2	3	0.274^f^
**Non pharmacological treatment**							
Corneal cross linking (CXL)	1	1	0	1.00^f^	0	1	0.32^f^
Surgical intervention	3^ g^	1	2	0.57^f^	3	0	0.54^f^
Both CXL and surgical	3^ h^	0	3	0.08^f^	0	3	0.02^f^
Total non pharmacological treatment	7 (24%)	2 (12.5%)	5 (38%)	0.19^f^	3 (20%)	4 (57%)	0.15^f^
Median BCVA (Snellen), first visit (25th percentile; 75th percentile)^b^	0.8 (0.08;1.0)	0.9 (0.4;1.0)	0.55 (0.02;0.9)	0.130^d^	0.85 (0.35;1.0)	0.04 (0.001;0.9)	0.041^d^
Median BCVA (Snellen), last visit (25th percentile; 75th percentile)^c^	1.0 (0.25;1.0)	0.95 (0.73;1.0)	1.0 (0.23;1.0)	0.854^d^	1.0 (0.9;1.0)	0.4 (0.075;0.925)	0.029^d^

a = 3 patients with no information on duration of symptoms (2 with monomicrobial growth, 1 with polymicrobial growth).

b = 1 patient with polymicrobial growth information missing on BCVA at first visit.

c = 2 patients with monomicrobial growth information missing on BCVA at last visit.

d = Mann–Whitney U-test, 2-tailed.

e = chi^2^ test, 2-sided.

f = Fisher’s exact test, 2-sided.

g = 2 patients received an amniotic membrane transplant, and 1 patient had a Gundersen.

h = 2 patients received amniotic membrane transplant and 1 patient later had the eye eviscerated.

BCVA = best corrected visual acuity.

Most of the patients (26/29; 90%) received topical treatment with fluoroquinolones. Of these, 18 patients received a fluoroquinolone antibiotic only, 15 patients were treated with levofloxacin (5 mg/mL). One of these patients also received additional ciprofloxacin ointment (3 mg/g) for the night as previously described above, and three patients received moxifloxacin (5 mg/mL). A combination of a fluoroquinolone antibiotic and an additional topical antibiotic was given to 8 patients. Of these, three patients received levofloxacin (5 mg/mL) in combination with chloramphenicol as ointment (10 mg/g). Two patients received levofloxacin (5 mg/mL) in combination with fusidic acid as ointment (1%); one patient received levofloxacin (5 mg/mL) in combination with fortified vancomycin drops (50 mg/mL); one patient received levofloxacin (5 mg/mL) in combination with tobramycin (3 mg/mL) and one patient received moxifloxacin (5 mg/mL) in combination with chloramphenicol as ointment (10 mg/g). The remaining three patients, not treated with a fluoroquinolone antibiotic as initial treatment, received a combination of fortified topical vancomycin (50 mg/mL) and ceftazidime (50 mg/mL).

Median duration of topical therapy was 15 days. There were no statistically significant differences in median duration of topical treatment between the group of patients with monomicrobial and polymicrobial growth (data not shown). Additional intervention such as corneal cross linking, amniotic membrane transplant, or evisceration was reported in 7/29 cases (24%) (Table 1). The eviscerated patient was an elderly patient with recurrent herpetic keratitis who had undergone a penetrating keratoplasty in the past with a best corrected visual acuity of 0.01 Snellen decimals prior to the episode of infectious keratitis.

In 23 of the 29 patients, the isolated *C. macginleyi* strains were stored; one of these isolates was recovered very late and therefore not subjected to whole-genome sequencing. There were no statistically significant differences between patients with monomicrobial (n = 16) and polymicrobial growth (n = 13) regarding any of the background characteristics, best corrected visual acuity at the first or last visit, or treatment (Table 1).

All tested *C. macginleyi* isolates were susceptible to benzylpenicillin (n = 25), vancomycin (n = 25), gentamicin (n = 25), ciprofloxacin (n = 27), and moxifloxacin (n = 23). Breakpoints for chloramphenicol, ceftazidime, and levofloxacin are currently lacking. MIC determination for chloramphenicol was performed for 23 strains of *C. macginleyi*; six had a MIC of ≤ 3 mg/L, 16 had MIC values ranging from 4 to 8 mg/L, and one had a MIC of 128 mg/L. MIC determination for ceftazidime was performed in 22 strains; 19 had a MIC of ≤ 8 mg/L and 3 had a MIC of 12 to 24 mg/L. Low MIC values (≤ 0.1 mg/L) were seen for levofloxacin in all 23 isolates tested (Table 2).

**Table 2:** Antibiotic susceptibility pattern of *Corynebacterium macginleyi* strains isolated from patients with infectious keratitis, determined as minimum inhibitory concentration.

Strain ID	Benzylpenicillin S ≤ 0.125 mg/L R > 0.125 mg/L (mg/L)	Chloramphenicol no breakpoint currently available	Vancomycin S ≤ 2 mg/L R > 2 mg/L (mg/L)	Ceftazidime no breakpoint currently available	Levofloxacin no breakpoint currently available	Gentamicin S ≤ 1 mg/L R > 1 mg/L (mg/L)	Ciprofloxacin S ≤ 1 mg/L R > 1 mg/L (mg/L)	Moxifloxacin S ≤ 0.5 mg/L R > 0.5 mg/L (mg/L)
171203	0.012	4	0.5	8	0.047	0.064	0.032	0.032
14T631	0.032	4	0.75	4	0.047	0.094	0.023	0.023
160806	0.023	5	0.75	2	0.032	0.125	0.023	0.016
171015	0.032	4	0.75	24	0.064	0.125	0.125	0.032
160812	0.023	4	0.5	3	0.047	0.094	0.023	0.032
150801	0.016	4	0.5	3	0.047	0.094	0.094	0.023
14T514	0.008	6	0.75	1	0.094	0.064	0.047	0.032
160811	0.016	6	0.75	4	0.064	0.125	0.064	0.032
151011	0.023	3	0.5	16	0.047	0.064	0.023	0.023
180216	0.016	4	0.75	8	0.047	0.125	0.032	0.023
12T220	0.016	6	0.5	6	0.047	0.125	0.023	0.023
180208	0.06	6	0.75	1	0.047	0.064	0.032	0.023
14T168	0.023	8	0.75	12	0.064	0.19	0.032	0.032
12T66	0.012	1	0.5	3	0.023	0.38	0.023	0.016
9T245	0.008	4	0.5	1.5	0.064	0.032	0.016	0.016
160430	0.016	2	0.5	4	0.032	0.032	0.016	0.008
170718	0.008	1	0.5	2	0.016	0.016	0.012	0.016
180126	0.006	2	0.75	0.5	0.023	0.125	0.023	0.016
06T638	0.023	128	0.75	8	0.032	0.064	0.023	0.032
14T424	0.003	4	0.75	1.5	0.032	0.047	0.012	0.012
160603	0.016	6	0.75	8	0.047	0.19	0.023	0.023
161211	0.012	4	0.75	3	0.047	0.38	0.064	0.047
*	0.016	1.5	0.5	1.5	0.047	0.094	0.016	0.023
**Susceptibility testing performed according to prevailing routine at the time of the disease episode. Information retrieved from the microbiology report in the medical journal since isolates not stored**								
						0.125	0.064	
			0.25			0.064	0.023	
	0.004	0.75	0.38		0.023		0.032	
	0.008		0.5				0.032	0.032

*Stored but retrieved very late therefore not sequenced.

In 2 patients susceptibility testing was not performed at the time of disease episode, and later determination was not possible since the isolates were not stored or were too slow-growing.

### Whole-genome sequencing of *C. macginleyi*

In total, 22 strains of *C. macginleyi* were genome sequenced. Table 3 presents the sequencing and genome statistics for each genome, including number of paired-end reads after quality filtering, average read length, number of contigs after assembly, coverage, total genome size, GC content, and GenBank accession number. The GC content was almost identical in each genome, ranging from 57.1 to 57.2%. Draft genome sizes varied between 2325 and 2484 kb (average: 2403 kb), giving a maximum size difference between genomes of 159 kb. GC content and genome sizes were in agreement with the three previously sequenced genomes of *C. macginleyi*^18^.

**Table 3:** *Corynebacterium macginleyi* genome sequences.

Strain	Sequence reads	Average read length	Coverage (fold)	Contigs	N50 (kb)	Size (kb)	GC content (%)	CDS	GenBankcession
150801	1,516,016	246	152	42	158	2436	57.1	2533	JAACCO
151011	1,841,548	245	192	59	156	2403	57.2	2502	JAACCN
160430	1,978,036	253	203	50	118	2436	57.1	2541	JAACCM
160603	2,674,252	247	258	124	38	2459	57.2	2623	JAACCL
160806	1,659,746	248	167	48	121	2,429	57.1	2518	JAACCK
160811	1,822,352	251	190	55	125	2374	57.1	2430	JAACCJ
160812	1,697,424	254	182	75	75	2325	57.2	2413	JAACCI
161211	2,079,840	250	215	56	125	2384	57.1	2466	JAACCH
170718	1,505,640	252	156	87	80	2383	57.2	2515	JAACCG
171015	1,866,726	254	196	51	114	2387	57.1	2451	JAACCF
171203	1,433,984	245	140	59	128	2394	57.2	2515	JAACCE
180126	1,379,894	247	141	54	107	2368	57.2	2442	JAACCD
180208	1,597,180	249	162	43	132	2428	57.1	2527	JAACCC
180216	1,291,684	252	135	47	175	2390	57.2	2489	JAACCB
06T638	1,457,578	253	150	49	168	2430	57.1	2510	JAACCA
9T245	1,914,450	253	191	78	82	2484	57.1	2662	JAACBZ
12T66	1,736,406	246	173	87	56	2411	57.2	2544	JAACBY
12T220	1,422,440	253	145	36	228	2443	57.1	2558	JAACBX
14T168	1,398,060	256	145	45	170	2434	57.1	2551	JAACBW
14T424	1,466,918	256	158	58	129	2338	57.2	2447	JAACBV
14T514	2,130,752	235	208	58	105	2387	57.2	2481	JAACBU
14T631	2,542,756	239	252	56	104	2351	57.2	2411	JAACBT
**Previously sequenced** ***C. macginleyi*** **genomes**									
CCUG 32361	n.d	n.d	96	56	128	2419	57.1	2553	REGE
NML 080212	n.d	n.d	95	77	58	2417	57.1	2535	REGD
NML 120205	n.d	n.d	109	40	163	2349	57.2	2400	REGC

The number of CDS predicted in the genomes by the RAST annotation pipeline ranged from 2411 to 2662 (average: 2505 CDS). About 37% of all CDS were annotated as hypothetical or conserved hypothetical proteins; this reflects the current lack of functional knowledge regarding ocular corynebacteria. A KEGG analysis could assign 45% (1179 CDS in strain 12T220) of all CDS to a KEGG pathway; among these, 20 CDS were assigned to lipid metabolism. A large number of repeat regions were found (87 regions in strain 12T220).

### Phylogenomic comparison of *C. macginleyi*

**Figure 1:**
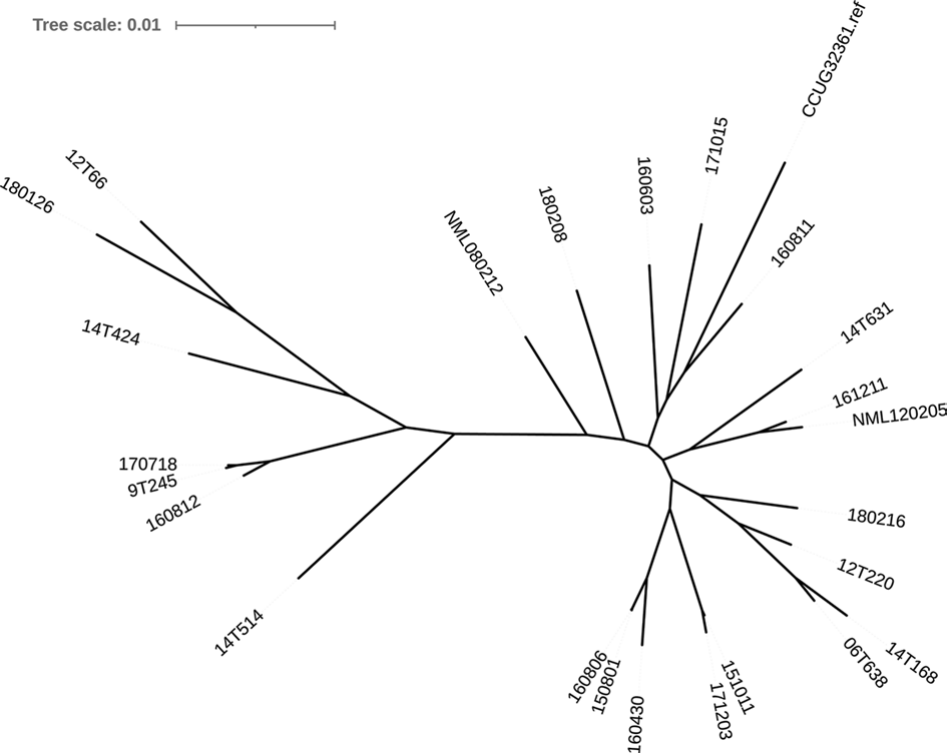
Phylogenomic comparison of all sequenced *C. macginleyi* strains. Results of a Parsnp analysis visualized with iTOL as an unrooted tree. The core genome covered 82% of the reference genome (CCUG32361); in total, 75,158 SNPs were found. The population could be divided into two clades: a major clade of 18 strains (clade I) and a minor clade of 7 strains (clade II). All strains were unique; the level of strain-specificity is illustrated by the length of the branches.

The genome sequences of the 22 strains and the three previously sequenced strains (Table 3) were phylogenetically compared by calling single nucleotide polymorphisms (SNPs) within the core genome. According to a Parsnp analysis, the core genome comprised 82% of the reference genome (strain CCUG 32361), with a total number of 75,158 SNPs in the core genome (Fig. 1). Each of the strains was individual; that is, they carried many SNPs. Even the closest relatives, strains 150801 and 160806, still had 563 SNPs in the core genome (comprising 91% of each genome). The SNP analysis further revealed that *C. macginleyi* strains could be separated into two main clades: a major clade (hereafter called clade I) containing 18 strains and a minor clade (clade II) containing 7 strains (Fig. 1). Subgrouping the patients according to the clade assignment of the isolated *C. macginleyi* strains revealed that patients with a clade II strain (n = 7) were significantly older than patients with a clade I strain (n = 15), with median ages of 71 years and 50 years, respectively (*p* = 0.002). Patients with a clade II strain had lower median best corrected visual acuity at the first visit (0.04 vs. 0.85; *p* = 0.041) and last visit (0.4 vs. 1.0; *p* = 0.029) than patients colonized with a clade I strain (Table 1).

**Figure 2:**
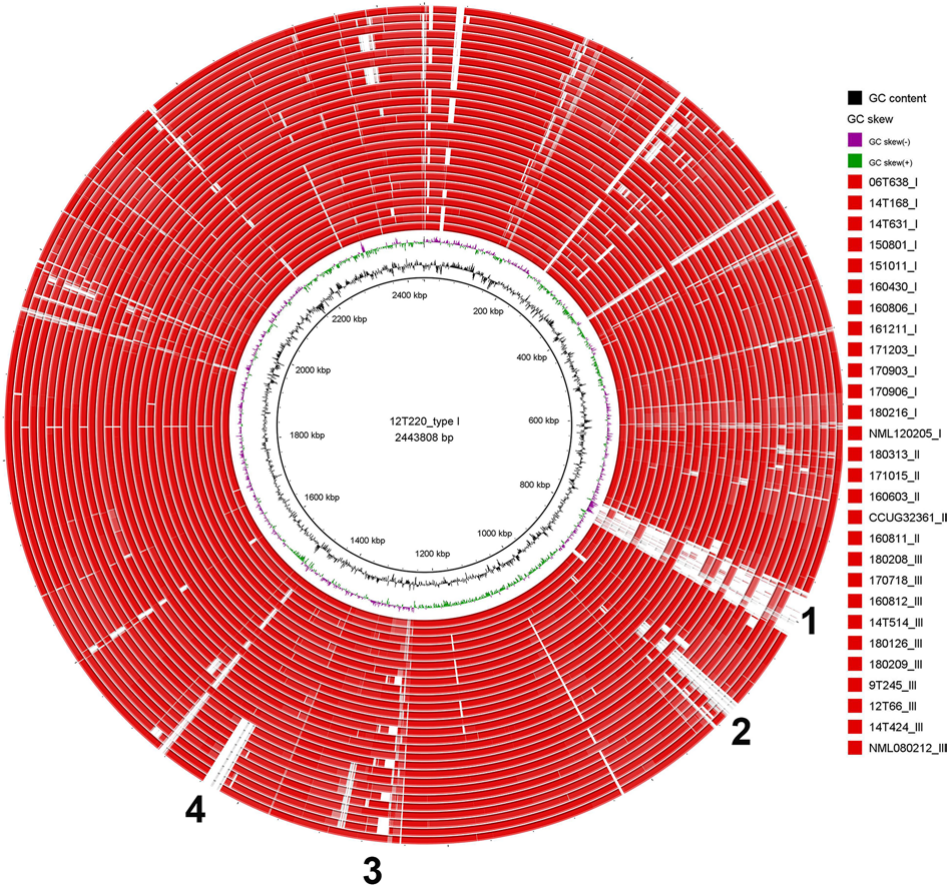
Visualization of the comparison of all *C. macginleyi* genomes. Strain 12T220 was taken as reference genome. The analysis revealed strong overall synteny. Only a few genomic islands were present, such as a prophage (labeled as 1), two islands that were absent in all clade II strains but present in most clade I strains (labeled as 2 and 4), and an island containing the CRISPR/cas gene cluster (labeled as 3).

In order to determine the presence of genomic islands, we conducted a total genome sequence comparison with BRIG (Fig. 2). The analysis revealed a strong synteny among the strains; only a few genomic islands were found, including two islands that were present in most clade I strains but missing in all clade II strains. One island of 18 kb was present in all clade I strains except NML 080212, encoding many hypothetical proteins, mobile element proteins, and an endonuclease. Another island of 14 kb was present in all clade I strains except NML 080212, CCUG 32361, and 180208, encoding hypothetical proteins and a few proteins associated with lipid metabolism, such as a glycerophosphodiester phosphodiesterase. The genomes of nine strains contained a 44 kb prophage that differed among the strains. A CRISPR/cas locus (type I-E) of 11 kb was present in 19 strains.

### Comparison with other corynebacterial species

**Figure 3:**
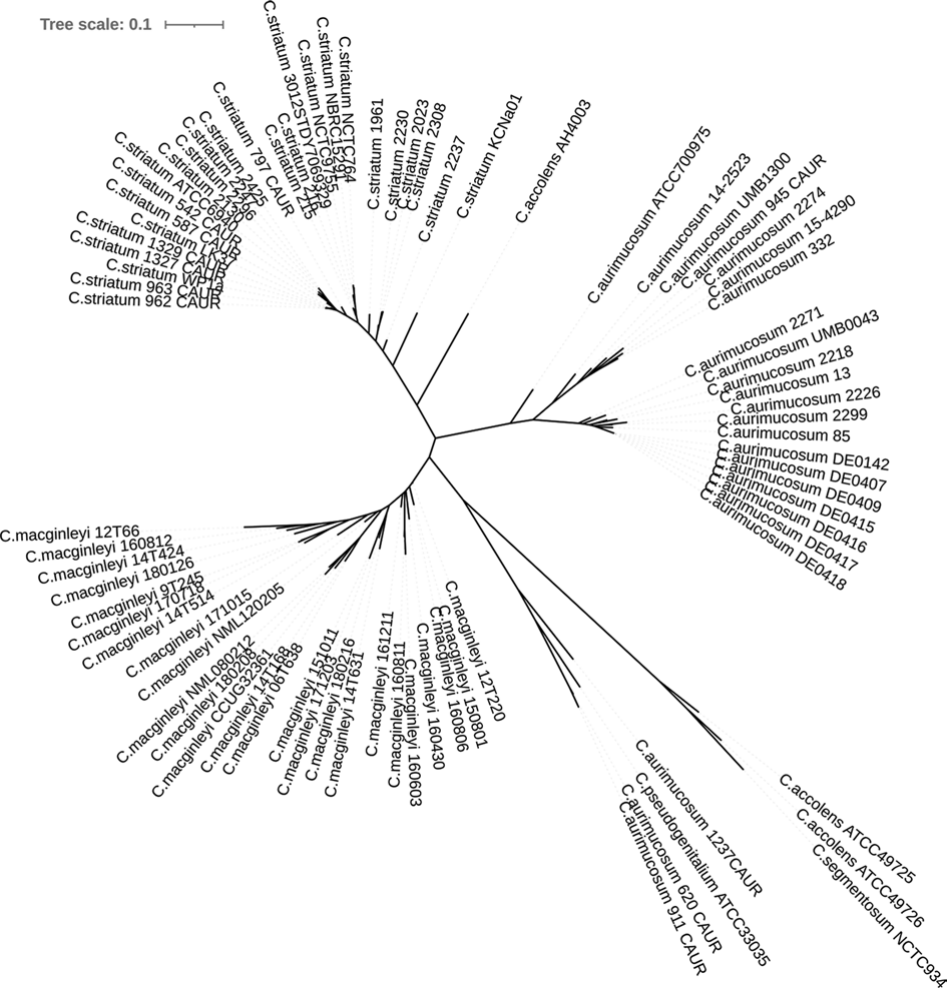
Phylogenomic comparison of *C. macginleyi* with corynebacterial relatives. Results of CSI phylogeny analysis visualized with iTOL as an unrooted tree. Around 80% of the genome of the reference strain (*C. macginleyi* 12T220) could be aligned to the genomes of *C. accolens* and *C. segmentosum*. According to an ANI analysis, *C. accolens* and *C. segmentosum* should be considered as one species; they had an ANI of > 95%. Only ca. 7% and ca. 6% of the reference genome could be aligned with the genomes of *C. aurimucosum* and *C. striatum*, respectively. A few mistakes in corynebacterial species assignments were uncovered: three strains assigned to *C. aurimucosum* (620_CAUR, 911_CAUR, 1237_CAUR) were wrongly classified into this species but actually belong to the species *C. pseudogenitalium*, and one strain assigned to *C. accolens* (AH4003) is a close relative of *C. striatum* (ANI of 83%) and should be renamed.

Comparative analyses showed that the closest relatives of *C. macginleyi* were *C. accolens* and *Corynebacterium segmentosum;* the latter two should be considered as one species as judged from average nucleotide identity (ANI) determination (Fig. 3). The ANI between *C. macginleyi* and the two species *C. accolens* and *C. segmentosum* was around 88% in both cases. Other related corynebacterial species were *Corynebacterium striatum* and *Corynebacterium aurimucosum*, each of which had an ANI of 73% with *C. macginleyi*. Three strains assigned to *C. aurimucosum* were wrongly classified into this species; they actually belonged to the species *Corynebacterium pseudogenitalium*, which had an ANI of 77% with *C. macginleyi*.

## Discussion

*C. macginleyi* was first described by Riegel et al.^1^. Until recently, infections caused by *C. macginleyi* have only been described as case reports, mainly of ocular infections^11,12^. With recent diagnostic developments such as MALDI-TOF MS, determination to species level has become faster, easier, cheaper, and hence more frequently performed. This offers new possibilities to identify corneal pathogens among bacteria previously considered as commensal and/or low-virulence.

In this article, we report clinical characteristics of bacterial keratitis presumably caused by *C. macginleyi* and genomic traits of *C. macginleyi* isolated from infected corneal ulcers. Contact lens wear was the most common risk factor, noted in 66% (19/29) of the cases. This high rate of contact lens wear in the present cohort may indicate that biofilm formation of the bacterial causative agent is involved, as suggested by both Ruoff et al.^11^ and Suzuki et al.^12^. The clinical course of infectious keratitis caused by *C. macginleyi* could generally be described as uncomplicated, with a median best corrected visual acuity of 0.8 Snellen decimals at the first visit and 1.0 Snellen decimals at the last visit and no need for corneal transplantation. This may be due to both the lack of virulence traits and the antibiotic susceptibility pattern of *C. macginleyi*. Indeed, genome sequencing of 22 strains of *C. macginleyi* confirmed the absence of virulence factors known for a few other corynebacterial species such as *Corynebacterium diphtheriae* and *Corynebacterium pseudotuberculosis.* However, the evaluation of the pathogenic potential of *C. macginleyi* is difficult due to the absence of functional studies and the fact that a large portion of the genome harbors genes that encode proteins with unknown function (around 37% of all CDS); this underlines the lack of knowledge concerning ocular corynebacteria.

Our study describes for the first time the population structure of *C. macginleyi* on the basis of extensive genome sequencing and subsequent comparisons. It was found that the species can be divided into two phylogenetically distant lineages, here called clade I and clade II.

Before our study, only three *C. macginleyi* strains were genome sequenced^18^: two (NML 080212 and NML 120205) which caused ocular infections in Canada^21^ and one (CCUG 32361) which was derived from a culture collection. All three strains belong to clade I*.* No previous study has reported information regarding clade II strains or patients infected with these strains. In the present study, patients infected with clade I and clade II strains had different clinical characteristics and visual outcomes, with those infected with clade II isolates seeming to have a more serious infection as judged from best corrected visual acuity at first and last visit and the need for more than one surgical intervention. We have no explanation for this; it might be due to the fact that these patients were considerably older, or it could be due to a small sample size. Several genomic differences exist between clade I and clade II strains. For example, clade I strains harbor a genomic island that encodes among other things, a glycerophosphodiester phosphodiesterase, which is a protein that catalyzes glycerophosphodiester hydrolysis. In *Haemophilus influenzae*, a homolog of this protein has been shown to contribute to adhesion and evasion of the host immune response^22^.

The isolates of *C. macginleyi* in our study were fully susceptible to fluoroquinolones, which were the primary treatment for uncomplicated cases of infectious keratitis during the study period of 2004–2018. This is in contrast to an earlier finding that Etest showed a high level of fluoroquinolone resistance which correlated to mutations in the quinolone resistance-determining region (QRDR) of the *gyrA* gene^23^. The sequenced strains in our study had no mutations in the QRDR of the *gyrA* gene, which is in line with their phenotypic susceptibility to fluoroquinolones. In addition, susceptibility testing to benzylpenicillin, chloramphenicol, vancomycin, ceftazidime, and gentamicin showed a very low rate of reduced susceptibility among tested isolates of *C. macginleyi.*

Almost half of the patients displayed polymicrobial growth, with growth of additional bacteria (such as *Staphylococcus aureus*, coagulase-negative staphylococci, *Moraxella catarrhalis*, and *Cutibacterium acnes*) or fungi. The lack of any statistically significant differences between the groups of patients with mono- and polymicrobial growth regarding either background characteristics or outcome parameters may support the notion that *C. macginleyi* was the causative agent of infectious keratitis in the cases reported in this study. However, we cannot exclude the possibility that the lack of any significant differences between the two groups was due to unknown factors or to the small sample size.

Due to the retrospective design of this study and the lack of routine storage of isolates, we also compared the group of patients whose isolates were stored and sequenced (n = 22) with those whose were not (n = 7). We could not find any statistically significant differences in outcome measures and clinical features (data not shown). This does not support a selection of more severe cases in the group of patients whose isolates were stored, but we cannot exclude the possibility that this lack of difference was due to a small sample size. Nevertheless, it is plausible that the 22 strains of *C. macginleyi* stored and sequenced in this study were a representative cohort of *C. macginleyi* isolates from patients with infectious keratitis.

One limitation of the present study is its retrospective design, which may have resulted in loss of cases and isolates, since not all isolates of *Corynebacterium spp.* and diphtheroid rods were determined to species level at the time of the disease episode and/or stored for later analyses. Despite the small sample size, this study includes to date the largest number of patients with infectious keratitis caused by *C. macginleyi*. These sequences of 22 genomes can provide a foundation for meaningful population studies and functional investigations of host-interacting properties and the pathogenic potential of *C. macginleyi*.

*C. macginleyi* can be considered a corneal pathogen. Despite a mostly uneventful disease course of infectious keratitis, whole-genome sequencing revealed two different clades of *C. macginleyi*, one of which was previously undescribed and was associated with a more severe disease course.

## Materials and methods

### Patient population

Patients with suspected infectious keratitis and a positive corneal culture displaying growth of *C. macginleyi* were identified by a search of the database at the Department of Laboratory Medicine, Clinical Microbiology, Örebro University Hospital, covering January 2004 to June 2018. The search criterion was corneal cultures with growth of *Corynebacterium* spp., diphtheroid rods, or *C. macginleyi*. As previously described^24^, an additional search for the diagnosis code for keratitis (H16.9 in the International Classification of Disease, version 10) was performed for the years 2004–2014. Identified patients were eligible for inclusion if corneal cultures were positive for *Corynebacterium* spp., diphtheroid rods, or *C. macginleyi*. Stored isolates of *Corynebacterium* spp. or diphtheroid rods were determined to species level by matrix-assisted laser desorption/ionization time-of-flight mass spectrometry (MALDI-TOF MS) (Microflex LT and Biotyper 3.1, Bruker Daltonik, Bremen, Germany). Of the 29 patients identified with growth of *C. macginleyi*, 23 had stored isolates; however, one isolate was recovered very late, and so only 22 isolates were included in the genomic analysis.

To describe the clinical appearance of infectious keratitis in this study, we applied the clinical criteria for microbial keratitis proposed by Stapleton et al.^20^: stromal infiltration with overlying epithelial defect in combination with at least one of lesion within or overlapping the central 4 mm of the cornea and/or uveitis and/or pain. For further analysis, the patients were divided into groups according to the presence or absence of additional microorganisms isolated from the corneal ulcer (polymicrobial growth), and according to the phylogenetic clade of the isolated *C. macginleyi* strains.

The study was approved by the Regional Ethical Review Board of Uppsala (refs: 2015/335 and 2015/335/2). The study was carried out in accordance with the regulations and guidelines stated in this approval. Due to the retrospective design concerning the human participation of the study, the Regional Ethical Review Board of Uppsala stated that written consent was not mandated.

### Corneal cultures

Corneal cultures were performed in accordance with the Swedish State of the Art Document on Infectious Keratitis caused by Bacteria, Yeast, and Protozoa^25^ (https://swedeye.org/wp-content/uploads/2010/02/2001-123-70.pdf), and can be considered a routine procedure. Corneal samples were obtained by both a cotton tipped applicator and a knife blade, and directly inoculated on gonococcal (GC) agar (GC Medium Base, Becton Dickinson, Sparks, MD, USA, supplemented with 1% BBL IsoVitaleX enrichment), blood agar (3.9% Columbia Blood Agar Base, Oxoid, Basingstoke, Hampshire, UK, supplemented with 6% defibrinated horse blood), and Sabouraud (SAB) agar (1.3% Agar No 2, Lab M, Heywood, Bury, UK; 4% D-Glucose, VWR, Leuven, Belgium; 1% Peptone, Becton Dickinson). For enrichment and isolation of anaerobes, corneal samples were also inoculated in enrichment broth (FAB) (2.97% fastidious anaerobic broth, Lab M, supplemented with 1% D-glucose, VWR).

All corneal cultures were registered and processed at the Department of Laboratory Medicine, Clinical Microbiology, Örebro University Hospital, as follows. GC agar plates were incubated in CO_2_ at 36 °C, blood agar plates and the FAB media were incubated in air at 36 °C, and SAB plates were incubated in air at 30 °C. The GC agar and blood agar plates were checked for growth on days 1 and 2; if no growth was detected, a final check was undertaken on day 7. The SAB plates and the FAB media were checked daily until day 7; if no growth was seen, the SAB plates were discarded. As soon as growth was detected in FAB media, or at day 7 if no growth was detected, the broth was subcultured on both a GC agar plate which was then incubated for 2 days in CO_2_ at 36 °C and a FAA plate (LAB 90 Fastidious Anaerobe Agar) 4.6% (w/v) (LAB M, Heywood, UK) supplemented with defibrinated horse blood, 5% (v/v) incubated under anaerobic conditions (10% H_2_, 10% CO_2_, 80% N_2_) at 37 °C for 5 days.

### Antimicrobial susceptibility testing

Stored isolates of *C. macginleyi,* kept at − 80 °C at the Department of Laboratory Medicine, Clinical Microbiology, Örebro University Hospital, were subcultured on GC agar plates for antibiotic susceptibility testing (n = 23). MICs for benzylpenicillin, chloramphenicol, vancomycin, ceftazidime, gentamicin, ciprofloxacin, levofloxacin, and moxifloxacin were determined by a gradient test Etest (bioMérieux, Marcy l’Etoile, France) according to the guidelines of the European Committee on Antimicrobial Susceptibility Testing (EUCAST) (www.eucast.org). The Etest was performed on GC agar plates incubated in 5% CO_2_ at 35 °C and read daily with an endpoint reading on day 3. In the six cases where isolates were not stored, the susceptibility testing could not be performed as described above. In four of these cases, information on antibiotic susceptibility patterns was retrieved from the microbiology report in the medical journal. Data in this report were determined at the time of the disease episode by a gradient test according to the guidelines of either EUCAST or the Swedish Reference Group of Antibiotic Issues (RAF) (www.srga.org). Information was not available for the remaining two cases because susceptibility testing was not performed at the time of the disease episode.

### Whole-genome sequencing

Genomic DNA isolation of 22 *C. macginleyi* strains was performed using the MasterPure DNA purification kit (Epicentre). Concentration and purity of the isolated DNA was first checked with a NanoDrop ND-1000 (Peqlab, Erlangen, Germany), and exact concentration was determined using the Qubit dsDNA HS Assay Kit as recommended by the manufacturer (Life Technologies GmbH, Darmstadt, Germany). Illumina shotgun libraries were prepared using the Nextera XT DNA Sample Preparation Kit and subsequently sequenced on a MiSeq system using the v3 reagent kit with 600 cycles (Illumina, San Diego, CA, USA) as recommended by the manufacturer. Quality filtering was done with version 0.36 of Trimmomatic^26^. Assembly was performed with version 3.13.0 of the SPAdes genome assembler software^27^, using an average of 1,746,076 paired-end reads (range: 1,291,684–2,674,252) with an average read length of 249 bp (range: 235–256). Version 2.2.1 of Qualimap^28^ was used to validate the assembly and determine the sequence coverage. The average coverage was 177-fold (range: 135–258). All genome sequences are stored in GenBank with the following accession numbers: strain 170718, JAACCG000000000; strain 171015, JAACCF000000000; strain 171203, JAACCE000000000; strain 180126, JAACCD000000000; strain 180208, JAACCC000000000; strain 180216, JAACCB000000000; strain 06T638, JAACCA000000000; strain 9T245, JAACBZ000000000; strain 12T66, JAACBY000000000; strain 12T220, JAACBX000000000; strain 14T168, JAACBW000000000; strain 14T424, JAACBV000000000; strain 14T514, JAACBU000000000; strain 14T631, JAACBT000000000; strain 150801, JAACCO000000000; strain 151011, JAACCN000000000; strain 160430, JAACCM000000000; strain 160603, JAACCL000000000; strain 160806, JAACCK000000000; strain 160811, JAACCJ000000000; strain 160812, JAACCI000000000; and strain 161211, JAACCH000000000.

### Annotation and phylogenomics

Gene prediction and annotation of all genomes were performed with RAST^29^, and functional annotation was performed with BlastKOALA^30^. For phylogenomic analyses, the core genome was identified and aligned with the Parsnp program from the Harvest software package^31^. Reliable core-genome SNPs identified by Parsnp were used for reconstruction of whole-genome phylogeny. CSI phylogeny and JSpeciesWS were used for interspecies comparisons with other corynebacterial species^32^. Phylogenetic trees were visualized using the Interactive Tree Of Life (iTOL; https://itol.embl.de/). The BRIG program^33^ was used for visualization and for comparative genome analyses including the three previously published genomes of *C. macginleyi*^18^: strain CCUG 32361 (GenBank accession number: REGE00000000), strain NML 120205 (REGC00000000), and strain NML 080212 (REGD00000000).

### Statistical analysis

The statistical analysis was conducted using version 25 of the IBM SPSS software package. Median age, median duration of symptoms prior to first visit, and median best corrected visual acuity in Snellen decimals at first and last visit were compared using the Mann–Whitney U-test, 2-tailed. Baseline characteristics such as sex, laterality, risk factors for infectious keratitis, and treatment were compared using Pearson’s chi-square test or Fisher’s exact test as appropriate, 2-sided. A significance level of p < 0.05 was chosen.

## Supplementary Information

Supplementary Information
