# Supplementary material for: Clinical and genomic features of Corynebacterium macginleyi-associated infectious keratitis
Source: Sci Rep. 2021 Mar 16;11:6015. doi: 10.1038/s41598-021-85336-w (PMC7966771; doi:10.1038/s41598-021-85336-w)
Supplement: Supplementary file 1 — Supplementary Information [file 41598_2021_85336_MOESM1_ESM.pdf]

Clinical and genomic features of *Corynebacterium macginleyi*-associated infectious keratitis

Susanna Sagerfors[a*], Anja Poehlein[b], Mastaneh Afshar[c], Birgitta Ejdervik Lindblad[a], Holger Brüggemann[c], Bo Söderquist[d]

[a]Department of Ophthalmology, Faculty of Medicine and Health, Örebro University, SE 70182 Örebro, Sweden

[b]Department of Genomic and Applied Microbiology, Institute of Microbiology and Genetics, University of Göttingen, Göttingen, Germany

[c]Department of Biomedicine, Aarhus University, Aarhus, Denmark

[d]Department of Laboratory Medicine, Clinical Microbiology, Faculty of Medicine and Health, Örebro University, SE 70182 Örebro, Sweden

*Address correspondence to Susanna Sagerfors, susanna.sagerfors@regionorebrolan.se

Table S1. Additional microorganisms determined in patients with infectious keratitis and their co-appearance with clade I or clade II *Corynebacterium macginleyi*

	Total (n=11)	Clade I (n=6)	Clade II (n=5)
Staphylococcus aureus	1	-	1
Coagulase-negative staphylococci	4	3	1
Moraxella catarrhalis	1	1	-
Corynebacterium spp.	1	-	1
Cutibacterium acnes	3	1	2
Unidentified filamentous fungus	1	1	-
